# A survey of digitized data from U.S. fish collections in the iDigBio data aggregator

**DOI:** 10.1371/journal.pone.0207636

**Published:** 2018-12-19

**Authors:** Randal A. Singer, Kevin J. Love, Lawrence M. Page

**Affiliations:** Florida Museum of Natural History, University of Florida, Gainesville, Florida, United States of America; Western Sydney University, AUSTRALIA

## Abstract

Recent changes in institutional cyberinfrastructure and collections data storage methods have dramatically improved accessibility of specimen-based data through the use of digital databases and data aggregators. This analysis of digitized fish collections in the U.S. demonstrates how information from data aggregators, in this case iDigBio, can be extracted and analyzed. Data from U.S. institutional fish collections in iDigBio were explored through a strictly programmatic approach using the ridigbio package and fishfindR web application. iDigBio facilitates the aggregation of collections data on a purely voluntary fashion that requires collection staff to consent to sharing of their data. Not all collections are sharing their data with iDigBio, but the data harvested from 38 of the 143 known fish collections in the U.S. that are in iDigBio account for the majority of fish specimens housed in U.S. collections. In the 22 years since publication of the last survey providing information on these 38 collections, 1,219,168 specimen records (lots), 15,225,744 specimens, 3,192 primary types, and 32,868 records of secondary types have been added. This is an increase of 65.1% in the number of cataloged records and an increase of 56.1% in the number of specimens. In addition to providing specimen-based data for research, education, and various outreach activities, data that are accessible via data aggregators can be used to develop accurate, up-to-date reports of information on institutional collections. Such reports present collections data in an organized and accessible fashion and can guide targeted efforts by collections personnel to meet discipline-specific needs and make data more transparent to downstream users. Data from this survey will be updated and published regularly in a dynamic web application that will aid collections staff in communicating collections value while simultaneously giving stakeholders a way to explore collections holdings as they relate to the institutions in which they are housed. It is through this resource that collections will be able to leverage their data against those of similar collections to aid in the procurement of financial and institutional support.

## Introduction

Natural history collections provide unparalleled sources of data for understanding biological diversity [1]. However, the full potential of these collections has not been achieved because the specimens and associated information are easily accessible only to researchers at their home institutions. The total information—the really big data in institutional collections, including by far the largest source of biodiversity occurrence data—are visible to an unacceptably small number of scientists and students, and mostly invisible to the general public.

Several efforts over the past few years have targeted the problem of data inaccessibility (see http://www.aibs.org/public-policy/biocollections.html), including the Global Biodiversity Information Facility (GBIF), which serves both observational data (without vouchered specimen documentation) and specimen data, and several projects specific to certain taxa (e.g., FishNet2 and VertNet). These projects have improved access by creating searchable databases, but they have not supported the process of digitization or they have been limited taxonomically.

The National Science Foundation’s Advancing Digitization of Biodiversity Collections (ADBC) Program has moved the effort forward substantially by supporting the digitization of specimen-based data. Funded projects are organized as Thematic Collections Networks (TCNs) of institutions digitizing data to address challenging research questions. The ADBC made its first awards in 2011 and, as of 2017, has funded 20 such digitization projects (http://www.idigbio.org/wiki/index.php/TCN_Resources). iDigBio, headquartered at the University of Florida, is the national coordinating center for ADBC, and through the iDigBio search portal (iDigbio.org), data and media for millions of biological specimens are available in electronic format for the research community, government agencies, students, educators, and the general public. As of 1 January 2018, 106 million specimen records and 22 million media records were searchable through iDigBio.

In addition to providing specimen-based data for research, education, and various outreach activities, the data accessible via data aggregators can be used to develop accurate and up-to-date reports of information on institutional collections. Such reports present collections data in an organized and accessible fashion and can guide targeted efforts by collections personnel to meet discipline-specific needs and make the data more transparent to downstream users. Through this information, individual collections can leverage their unique value through comparisons of their holdings to those of similar collections to aid in the strategic planning and procurement of financial and institutional support.

This analysis on digitized fish collections in the U.S. demonstrates how information from data aggregators, in this case iDigBio, can be extracted and analyzed to provide this type of information. Fish collections were chosen because of the availability of previous surveys on fish collections. Comparisons with previous surveys provide information on change over time as well as information on the content of collections.

Comprehensive surveys of institutional fish collections in the U.S. have been conducted twice before. The first survey was in 1976 by Collette and Lachner [2], and a second survey was conducted in 1995 by Poss and Collette [3]. The results of a third survey are reported herein, with a cross-collection comparison similar to that done by Poss and Collette [3]. In contrast to the earlier surveys, which relied on interviews of institutional staff, computational methods are utilized to explore collections data published in iDigBio (iDigBio.org) and to create a permanent web resource for visualizing the data.

## Materials and methods

Data were synthesized in R Studio [4] using the ridigbio package [5]. ridigbio is a programmatic interface to iDigBio’s search application programming interface (API) that allows processing of specimen records as native data structures in R. Using functions exported by the package, specimen record searches were returned as data frames that provided the foundation for the analytical framework used in this survey. The ridigbio package also provided access to summary and aggregate data API endpoints provided by iDigBio, such as recordset metadata, normalized field counts, and dates bucketed for histograms.

iDigBio’s APIs provide data in a standardized format called Darwin Core (http://rs.twdg.org/dwc). Darwin Core is a set of standards developed by the TDWG Biodiversity Information Standards working group (http://www.tdwg.org/)). These standards include a glossary of terms intended to facilitate the sharing of information about biological diversity through a set of community developed standards. Data contained in iDigBio include both raw and indexed data structures. Raw data are the verbatim data mobilized by collections and indexed data are structured by iDigBio to improve the speed of data retrieval and synthesis by data users. Exploratory data analysis techniques were used to investigate query results returned by iDigBio’s APIs. When query limits were reached with the ridigbio package (due to restrictions on the API), the iDigBio download system was used to retrieve query results. All scripts used in the analyses and visualizing of results were developed specifically for this project (Table 1).

**Table 1:** R packages used in inventory queries, analysis and visualization. Each script generated using a package is listed.

Package	Scripts in which package was used	Citation
ridigbio	aggregate-plots.R, collection-plots.R, identifying-collections.R, idigbio-data-source.R	[5]
CartoDB	cartoWrite.R	[6]
RColorBrewer	aggregate-plots.R	[7]
countrycode	aggregate-plots.R, collection-plots.R	[8]
devtools	cartoWrite.R	[9]
dplyr	aggregate-plots.R, collection-plots.R, identifying-collections.R, idigbio-data-source.R, preps.R, species.R	[10]
httr	aggregate-plots.R, collection-plots.R, identifying-collections.R, idigbio-data-source.R	[11]
jsonlite	aggregate-plots.R, collection-plots.R, counts.R, identifying-collections.R, idigbio-data-source.R, preps.R, species.R	[12]
plotly	aggregate-plots.R, counts.R, preps.R, species.R	[13]
plotrix	aggregate-plots.R, collection-plots.R	[14]
plyr	aggregate-plots.R, build-data-frame.R, collection-plots.R, counts.R,identifying-collections.R, idigbio-data-source.R, preps.R, species.R	[15]
progress	aggregate-plots.R, collection-plots.R, identifying-collections.R, idigbio-data-source.R	[16]
reshape2	aggregate-plots.R, collection-plots.R,	[17]
rworldmap	aggregate-plots.R, collection-plots.R	[18]
scales	aggregate-plots.R, collection-plots.R	[19]
tidyr	counts.R, preps.R, species.R	[20]

Collections data are published in aggregations which iDigBio defines as recordsets. A recordset may contain all or only a portion of the specimen records from an institutional collection, or it may contain specimen records from several institutional collections. The funding model in the ADBC program for TCNs encourages groups of institutions to digitize and mobilize data around a particular research theme, which results in many institutionally mixed recordsets. For example, a recordset mobilized by a funded project may consist of specimens from a particular geographic area and include specimens from several taxonomic collections (e.g., fishes and amphibians). Because of the various ways in which datasets are mobilized as recordsets, they often do not map directly to institutional collections. This complicates data extraction of taxon-specific datasets. A method for flagging recordsets containing fish specimens was developed in order to query these heterogeneous data efficiently. When the data were extracted for analysis on 08/18/2017, there were 106,159,754 specimen records in iDigBio contained within 1,671 recordsets.

A set of parameters for defining fish collections was developed in order to compile data on individual collection holdings using data aggregated by iDigBio. For the purposes of this analysis, a fish collection was defined as a collection of non-fossilized, preserved fish specimens with associated locality data and/or tissues preserved for genetic analysis. Specimen records that did not meet these criteria were excluded (i.e., living stocks, collections labeled as “fish collection” but contained no fish records, specimens without data, etc.

**Fig 1:**
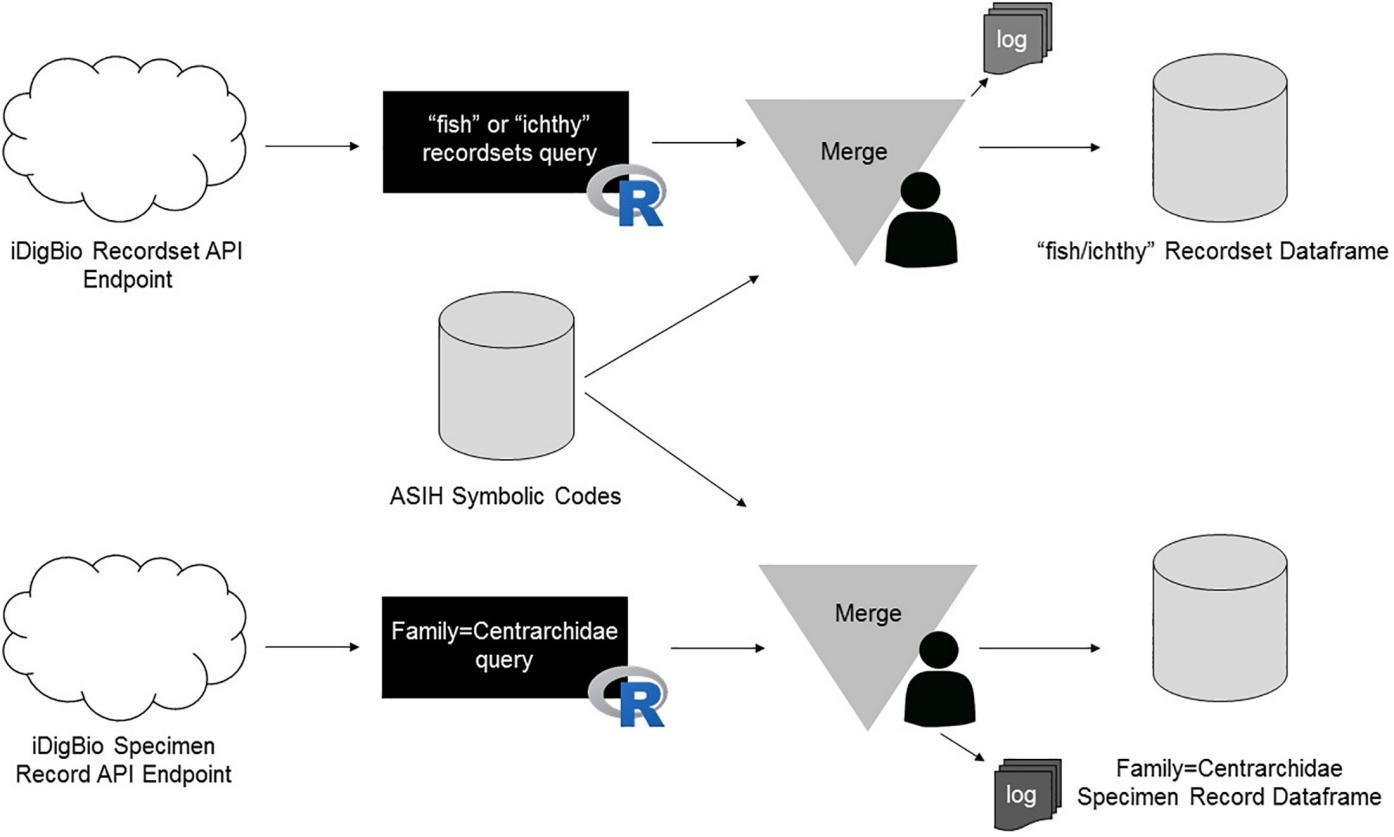
First approach workflow for discovering fish collections. Searches were run using the terms “fish” and “ichthy” alongside recordsets containing “Family = Centrarchidae” contained in iDigBio published recordsets.

Our approach investigated records using two distinct methodologies [https://github.com/melanostomias/rstuff/blob/master/scripts/identifying-collections.R] (Fig 1). The first involved looking for collections via their recordset metadata. These are properties of the dataset that have been included by the data publisher to specify the scope and ownership of the data [21]. Recordsets with “fish” or “ichthy” in their metadata were investigated. Institutional codes (e.g., USNM, ANSP), often included in recordset metadata or specimen records, could not be used to identify collection datasets because these codes were not specific to fish collections and often returned recordsets for collections other than fishes.

The second approach involved performing a specimen query of all iDigBio specimen data using the ridigbio package. Using the search parameter “Family = Centrarchidae”, a data frame was generated. These data were aggregated by: unique collection code, institution code and recordset identifier. Centrarchidae was selected because it is a common family that could be assumed to be present in every fish collection in the U.S.

The institution codes and collection codes found by the two methods were reconciled against the list of ASIH Standard Symbolic Codes for Institutional Resource Collections in Herpetology & Ichthyology [22], which may give either the institutional or collection code, on the American Society of Ichthyologists and Herpetologists (ASIH) website. Data belonging to these collections were identified and linked to the appropriate ASIH symbolic code and a checklist of collections data was created. Properties of records (e.g., recordset universally unique identifier (uuid)) were used to identify records within the checklist, rather than their institution code or collection code. This was done to identify collections that were not on the ASIH standardized list or collection records that were published as a part of another recordset that was not associated with its parent institution. In addition, institution codes and collection codes were not reported consistently by data providers. Some records are not discoverable by their collection or institution code due to a lack of standardization in the mobilized data (e.g., collection codes with abbreviations that did not provide any information about the collection type).

**Fig 2:**
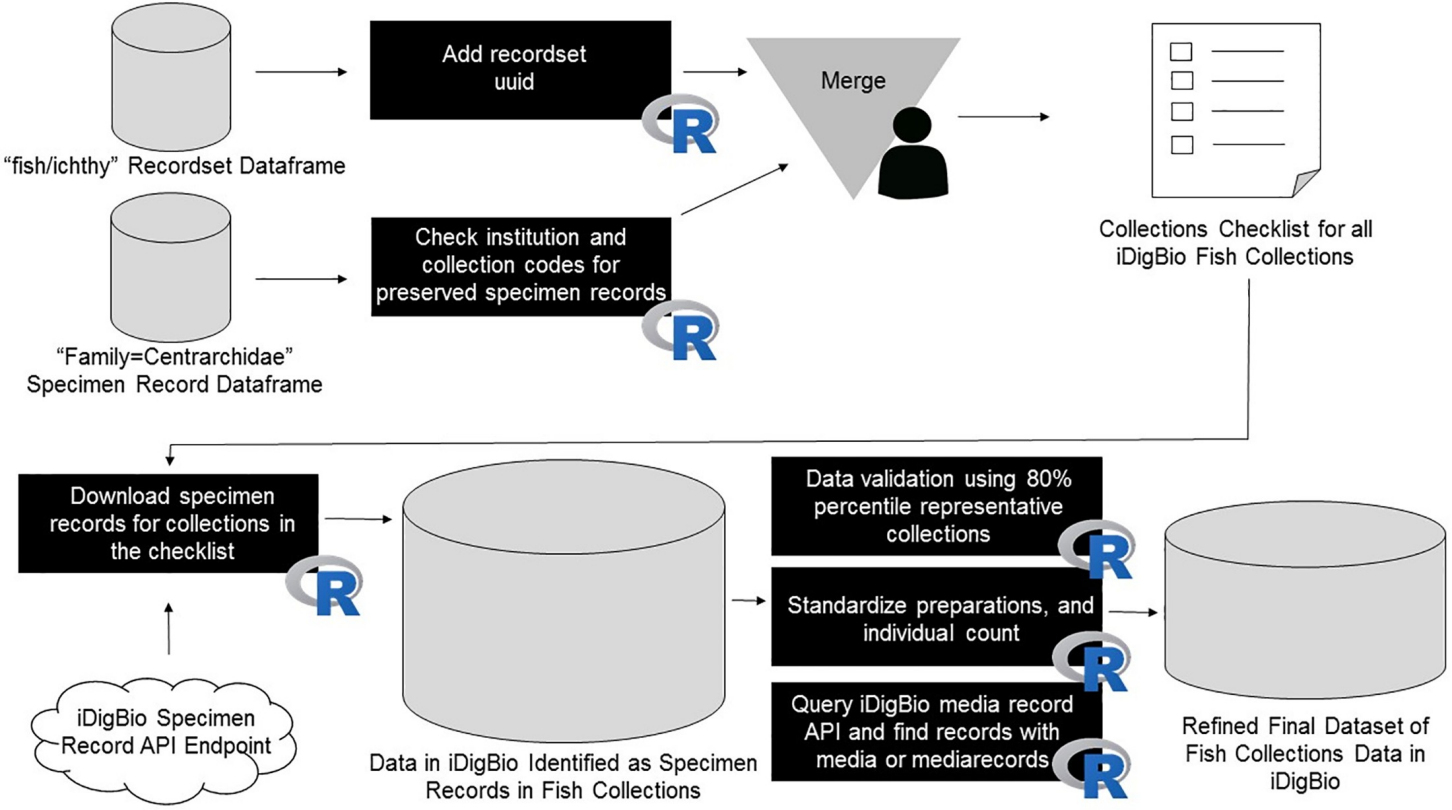
Second approach workflow for discovering fish collections. This process involved refining fish collection query results and then merging the outputs into a combined and standardized dataframe.

The first task for exploring the specimen data that met the criteria for a fish collection involved performing data validation [https://github.com/melanostomias/rstuff/blob/master/scripts/build-data-frame.R]. This process included comparing the entire dataset against specimen records in the 80^th^ percentile of three criteria determined to be representative of the entire dataset. These criteria included the following: quantity of published records, cardinality of families, and quantity of records identified as type material. Collection records meeting these criteria were then used to establish a dictionary of dwc:family data that was used to filter out erroneous data (e.g., reptile, amphibian, mammal or bird records mixed with fish records) while keeping fish records with misspellings and invalid nomenclature (Fig 2).

The analysis of the fish collections dataset required standardization or normalization of the data found in the variables "dwc.preparations" [https://github.com/melanostomias/rstuff/blob/master/scripts/preps.R] and "dwc.individualCount" [https://github.com/melanostomias/rstuff/blob/master/scripts/counts.R] in the specimen dataset. Although the Darwin Core standards attempt to provide reference definitions and examples for these terms, further data processing was required to perform comparative analyses. For "dwc.preparations", regular expressions were used to lump similar values together. Records lacking data in "dwc.individualCount" were isolated and regular expressions were used to parse any counts of individual specimens contained in "dwc.preparations" (e.g., “EtOH -15, Skeleton—1” = 16 individual specimens). Then, the synthesized counts of individual specimens from “dwc.preparations” and "dwc.individualCount" were standardized and summarized. The individual specimen counts for each collection were also plotted. No processing or data cleaning were performed on "dwc.scientificName" [https://github.com/melanostomias/rstuff/blob/master/scripts/species.R] (which is a determination to the most deducible taxonomic unit possible), and the values found in the raw specimen data were extracted and summarized. The associated cardinality (number of unique elements within each grouping) was plotted for each collection.

Additionally, informed decisions were made on using raw data versus iDigBio indexed data. In some cases raw data were more useful for reporting on a particular value due to the need to observe subtle nuances in the data (e.g., individual count). iDigBio indexed data were more useful for comparisons across the larger dataset with values that are more concrete (e.g., countries via ISO country code).

Data files and code were uploaded to a Dryad repository [23], cloned to a web server and a Shiny [24] web application was developed (http://www.fishfindR.net) to explore the queries employed in the survey interactively. The web application was organized into three major sections: a summary page that features a map generated using CartoDB [6][7], an individual collection data page, and a comparisons page with interfaces accessible to the broader community [25]. See S1 Appendix for a full list of collection names and acronyms used.

## Results

Poss and Collette [3] surveyed 118 fish collections, with 18 collections having 1 million or more specimens. The present analysis only focused on digitized fish collections aggregated by iDigBio, which contains data for 38 fish collections (S1 Appendix), 10 of which have over 1 million specimens and all of which were included in the Poss and Collette survey. This survey returned 3,107,368 specimen records (typically referred to in institutions as “lots”) with 44,128,165 specimens, a mean of 14.2 specimens per record. The analysis returned 1,005 unique family name entries containing at minimum 28,742 unique species entries. The fields referred to as “unique family name” and “unique species name” were taxonomic distinctions assigned to records, but no judgements were made on correct spelling or current validity of the name. Although many of these are misspelled, outdated or have errors in the text (e.g., the Catalog of Fishes [26] recognizes only 565 family names as valid), the names were accepted as presented by institutions. Numbers of families per institution were not reported by Poss and Collette [3], presumably because the surveyed collections did not include them. Records were available from every continent and 213 countries. The following countries were the most frequently sampled by ISO country codes [8]: U.S.A. (66.2%), Mexico (3.6%), Philippines (2.5%), Brazil (2%) and Venezuela (1.7%).

When compared with the report from Poss and Collette [3], there was a noticeable overall increase in holdings across the 38 collections sampled in both surveys. Significant growth was found for cataloged records (65.1%) and for cataloged specimens (56.1%) across all collections. Since the last survey, 1,219,168 specimen records, 15,225,744 specimens, 3,192 primary types, and 32,868 records of secondary types were added.

Of the 118 collections surveyed by Poss and Collette [3], nine were Canadian. Of the 109 U.S. collections, eight are no longer supported as separate biodiversity collections and their specimens have been transferred to another institution. In all cases, specimens were transferred to a nearby large regional collection with more than 50,000 specimen records [22]. This leaves 101 extant fish collections in the U.S. that participated in the Poss and Collette survey. The ASIH Standard Symbolic Codes for Institutional Resource Collections [22] lists 143 U.S. fish collections, of which 38 have mobilized records aggregated by iDigBio. Of the 105 collections that have not had their data aggregated by iDigBio, 22 (20.2%) were affiliated with an institution that mobilizes data aggregated by GBIF, and 6 (5.7%) mobilize data aggregated via FishNet2 or VertNet. Additionally, 4 (3.8%) were affiliated with an institution that does not share data through a data aggregator, but for which collections’ metadata are discoverable through the Global Registry of Biodiversity Repositories (GRBio). Five (4.8%) collections that were not sharing data via iDigBio mobilized their data through more than one other aggregator. Eighty-two (78.1%) collections were not discoverable through any data aggregator. Of the 143 known fish collections in the U.S., 62 (43.5%) share or have institutional support for sharing their data through a data aggregator. This value was determined by observing mobilized recordsets from other collections within the same institution, indicating institutional infrastructure to facilitate data sharing.

Poss and Collette [3] reported the following for each collection: numbers of lots, species, specimens, primary and secondary types, and visitors, growth in number of specimens, loan and exchange activity, accessioning activity, cataloging activity, available floor space, species holdings, computerization (percent collection databased), museum personnel, students receiving degrees, grants, and collection funding. For the present analysis, the following data were synthesized: numbers of records (lots), specimens, primary and secondary types, unique species and family name entries; methods of preparation, percent collection holdings by ISO country codes, and percent of records with an associated geopoint (latitude/longitude coordinates). Information on the educational and community services such as loans and number of students trained were omitted because they could not be synthesized from collection records. For each of the categories inventoried, the top five for each are listed below with an associated figure showing data for all collections.

**Fig 3:**
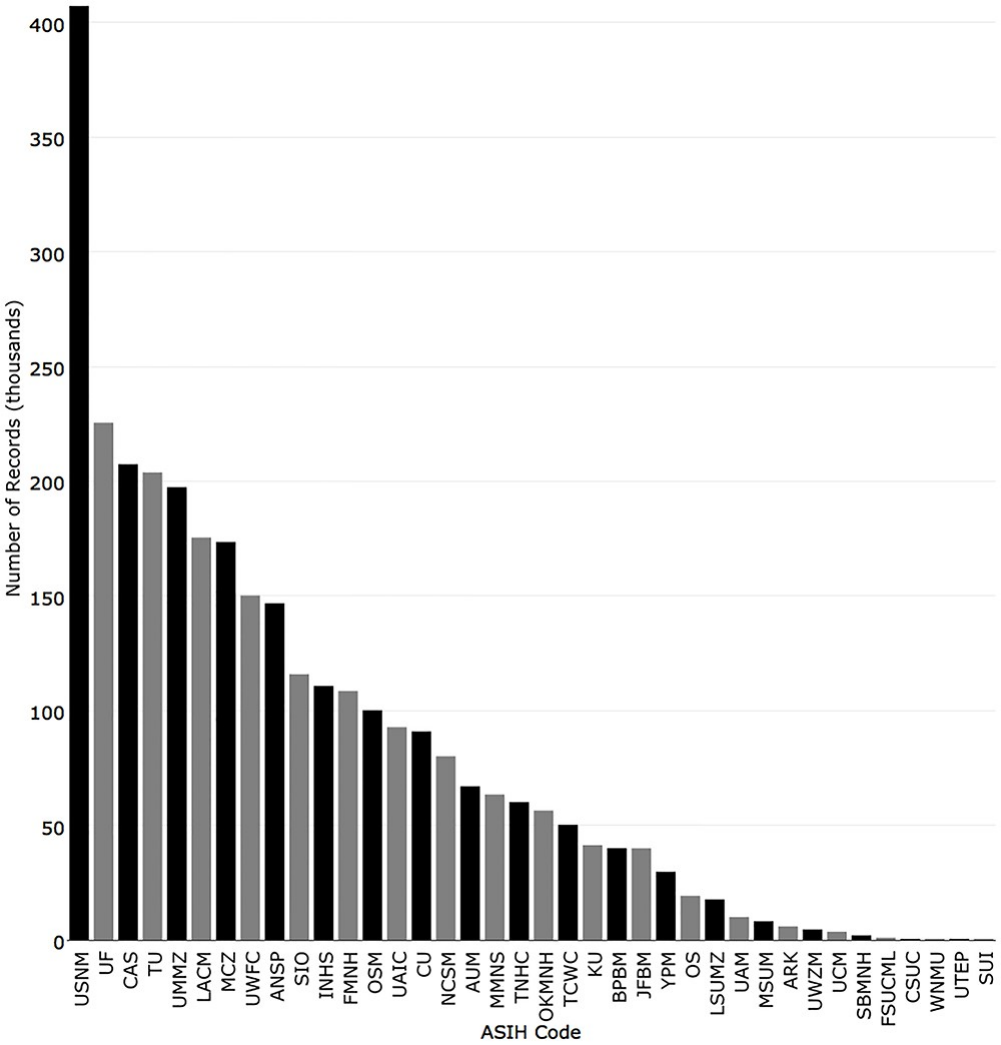
Number of specimen records (lots) returned for the 38 fish collections with recordsets in iDigBio.

The National Museum of Natural History had the most specimen records (407,231), followed by Florida Museum of Natural History (225,524), California Academy of Sciences (207,444), Tulane University (203,846), and University of Michigan (197,376) (Fig 3). The top 13 institutions each had over 100,000 records.

**Fig 4:**
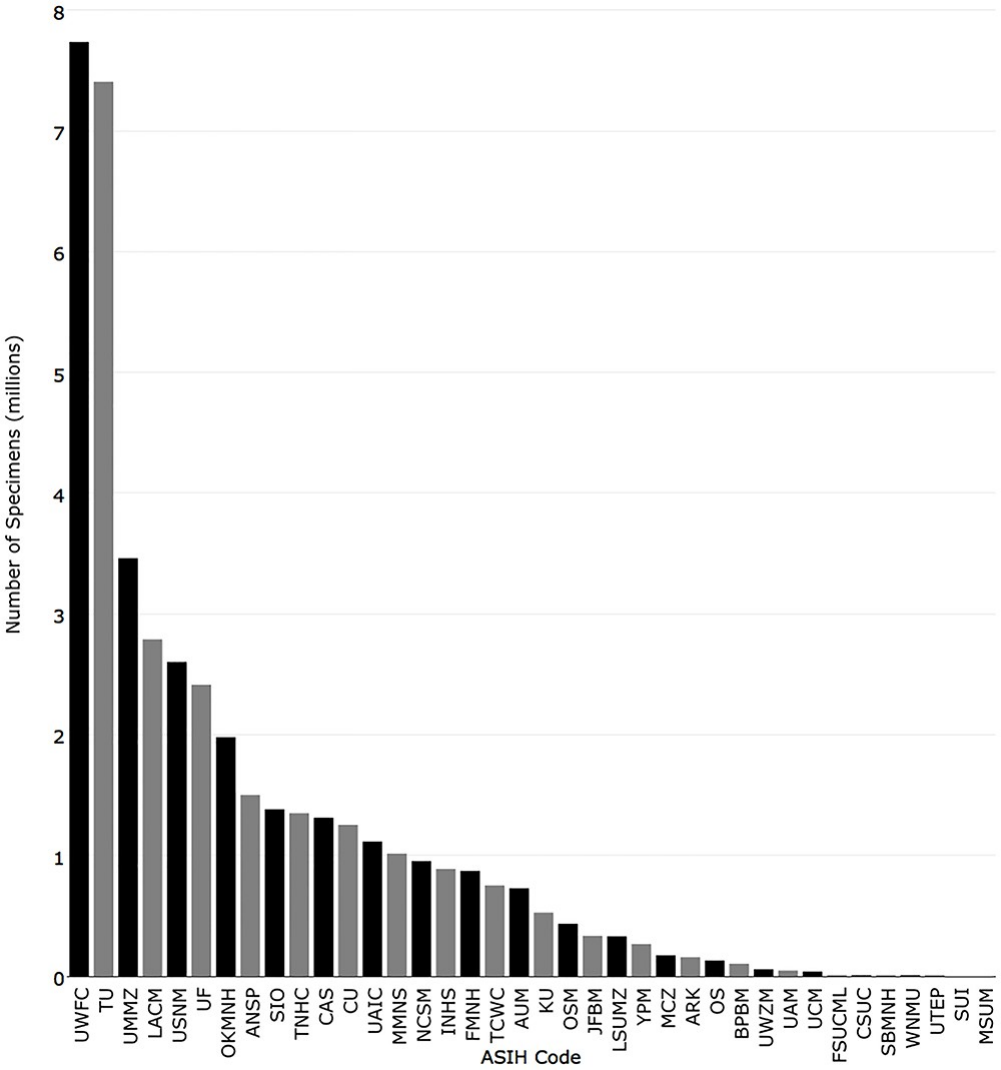
Number of individual specimens returned for the 38 fish collections with recordsets in iDigBio.

The University of Washington had the most specimens with 7,737,341, followed by Tulane University (7,407,714), University of Michigan (3,461,252), Natural History Museum of Los Angeles County (2,790,511), and National Museum of Natural History (2,603,610). The top 7 institutions had over 2 million specimens. (Fig 4).

**Fig 5:**
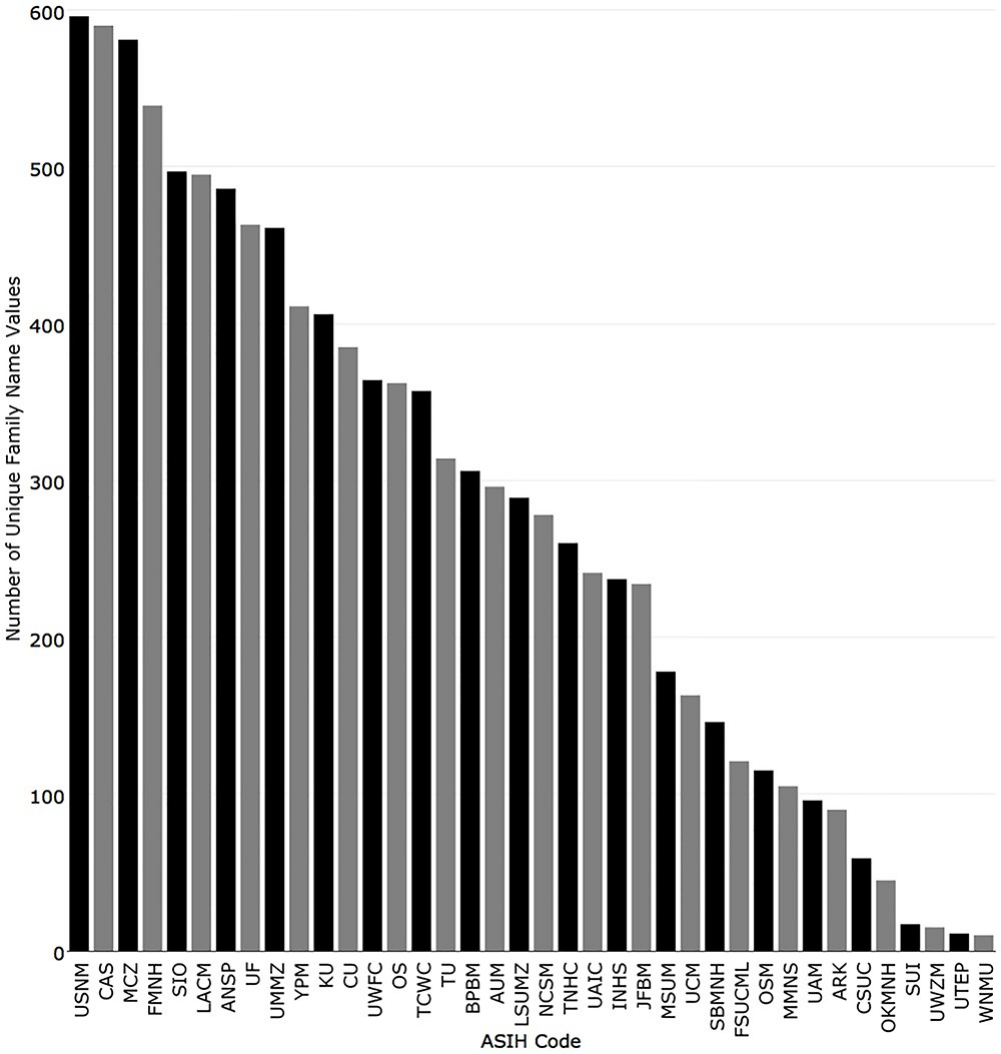
Number of unique family name values in records returned for the 38 fish collections with recordsets in iDigBio.

The National Museum of Natural History had the most unique family names (596), followed by California Academy of Sciences (590), Museum of Comparative Zoology, Harvard University (581), Field Museum of Natural History (539), and Scripps Institution of Oceanography (497) (Fig 5).

**Fig 6:**
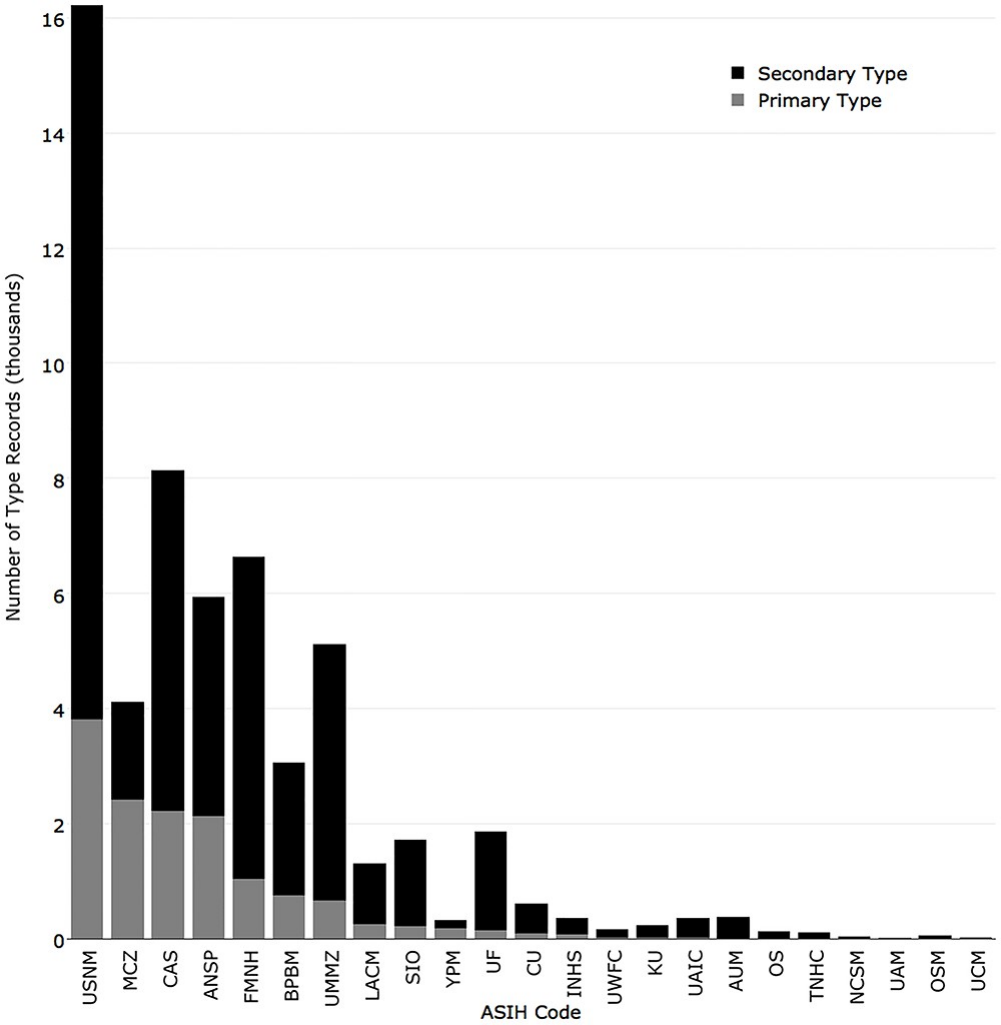
Number of type records returned for the 38 fish collections in iDigBio. Primary types are in light grey and secondary types are in black.

Of the 14,052 primary type specimens held by the 38 collections surveyed, National Museum of Natural History had the most (3,817), followed by Museum of Comparative Zoology, Harvard University (2,407), California Academy of Sciences (2,217), Academy of Natural Sciences of Drexel University (2,132), and Field Museum of Natural History (1,045) (Fig 6).

**Fig 7:**
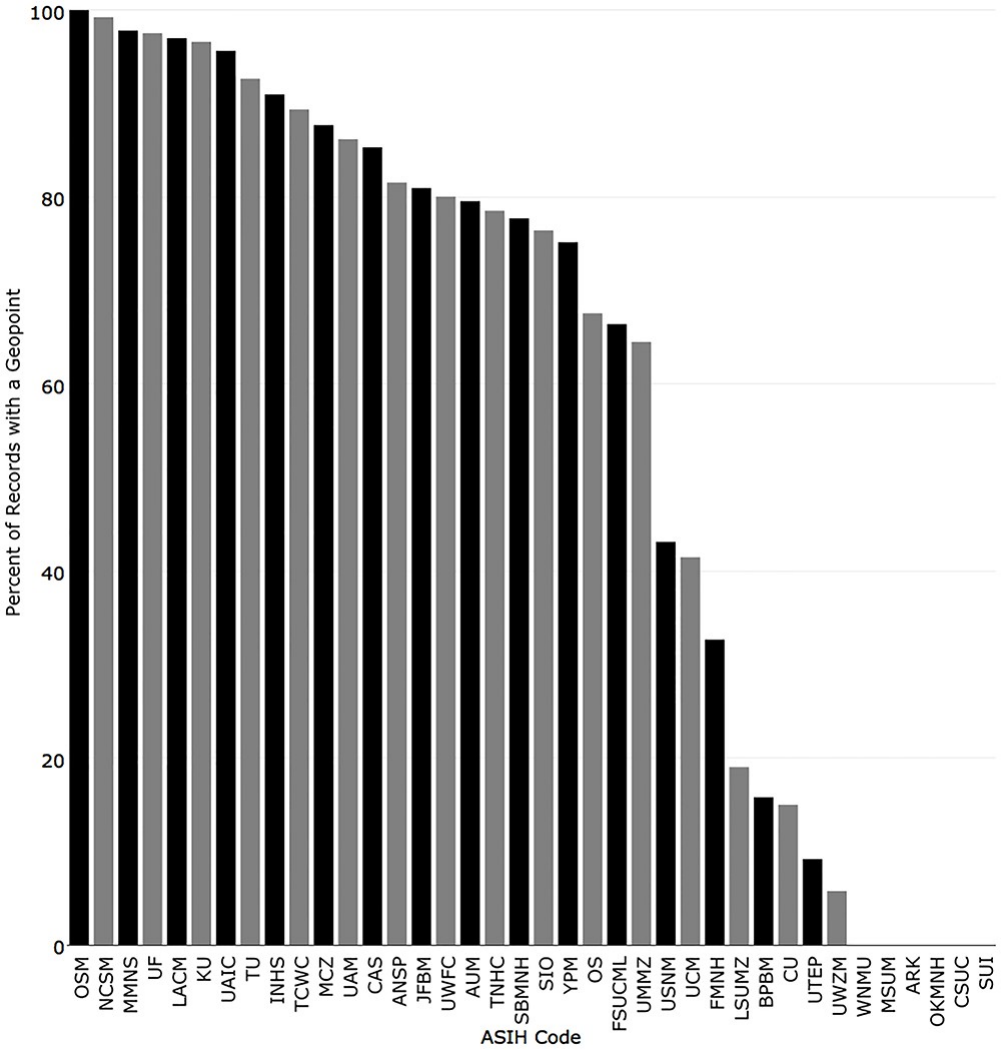
Percentage of records returned for the 38 fish collections in iDigBio with a geopoint.

Of the 42,776 secondary type specimen records in the 38 collections, National Museum of Natural History had the most (12,408), followed by California Academy of Sciences (5,915), Field Museum of Natural History (5,586), University of Michigan (4,459), and Academy of Natural Sciences of Drexel University (3,803) (Fig 7).

Twenty-four of the 38 institutions with the iDigBio indexed term “geopoint,” reported over 50% of their records with geopoints, with 16 of those being over 80%. For the 14 collections sharing less than 50% of their records with geopoints, five reported under 20%, and six were not sharing geopoints (Fig 7).

**Fig 8:**
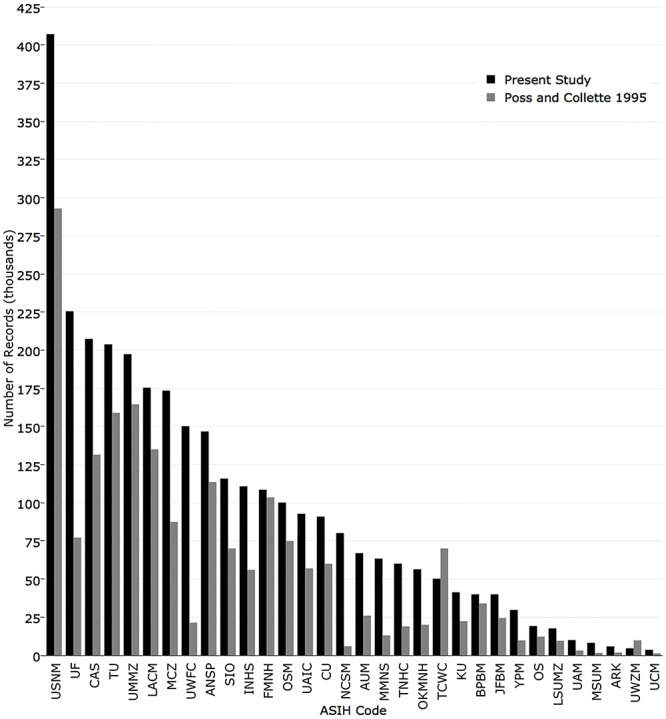
Comparison of the number of records published in iDigBio (black) with the number reported in Poss and Collette (1995) (grey).

To quantify change since the last survey, the values from this analysis were compared to those reported in Poss and Collette [3]. In 1995, the five institutions with the most cataloged records (lots) were National Museum of Natural History (292,932), University of Michigan (164,557), Tulane University (159,000), Natural History Museum of Los Angeles County (135,000), and California Academy of Sciences (131,500). The institutions with the greatest change were Florida Museum of Natural History (+148,374), University of Washington (+128,743), National Museum of Natural History (+114,299), Museum of Comparative Zoology, Harvard University (+86,035), and California Academy of Sciences (+75,944) (Fig 8).

**Fig 9:**
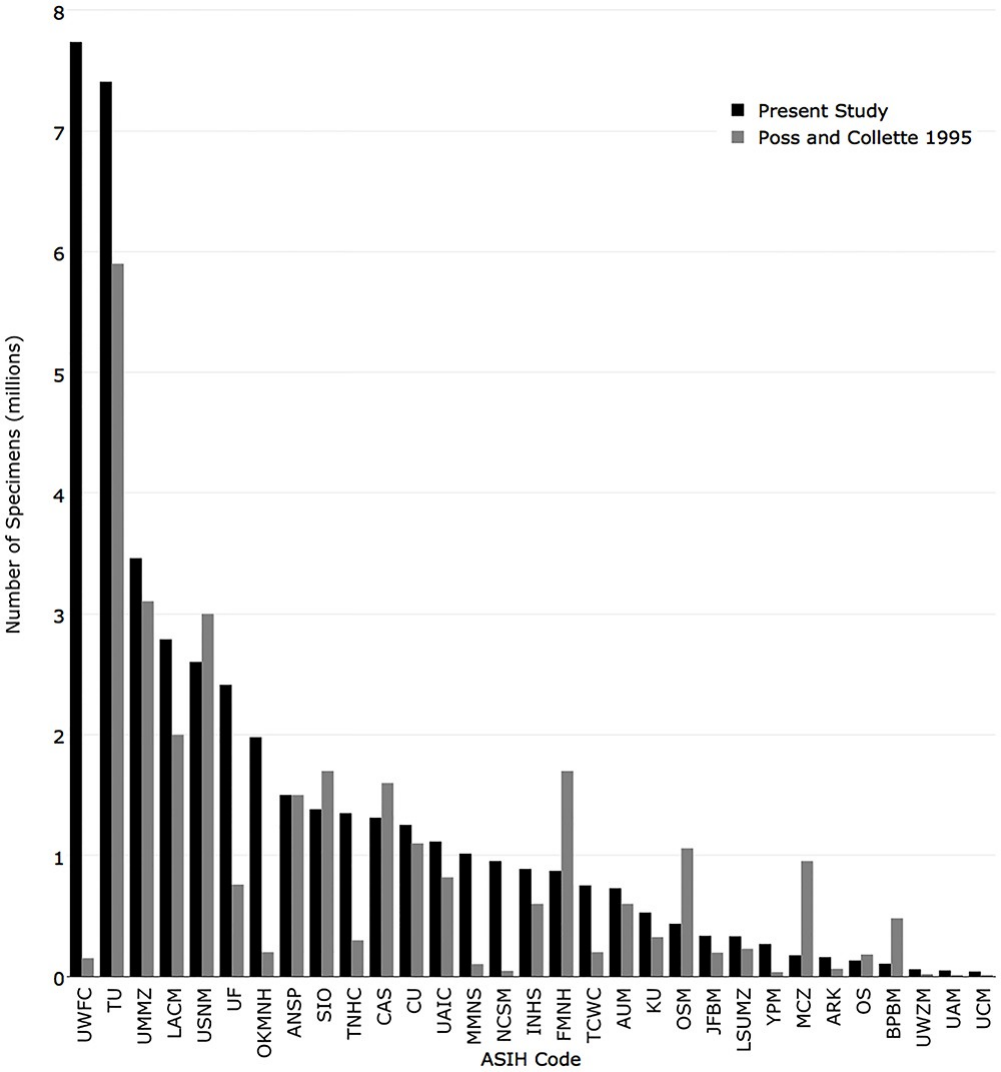
Comparison of the number of individual specimens currently published in iDigBio (black) with the number reported in Poss and Collette (1995) (grey).

In 1995, the five institutions with the most cataloged specimens were National Marine Fisheries Service La Jolla Laboratory (6,000,000), Tulane University (5,900,000), University of Michigan (3,104,903), National Museum of Natural History (3,000,000), and Natural History Museum of Los Angeles County (2,000,000). The institutions with the greatest change from 1995 were University of Washington (+7,587,341), Same Noble Museum (+1,778,306), Florida Museum of Natural History (+1,654,801), Tulane University (+1,507,714), and Texas Natural History Collections (+1,053,612) (Fig 9).

**Fig 10:**
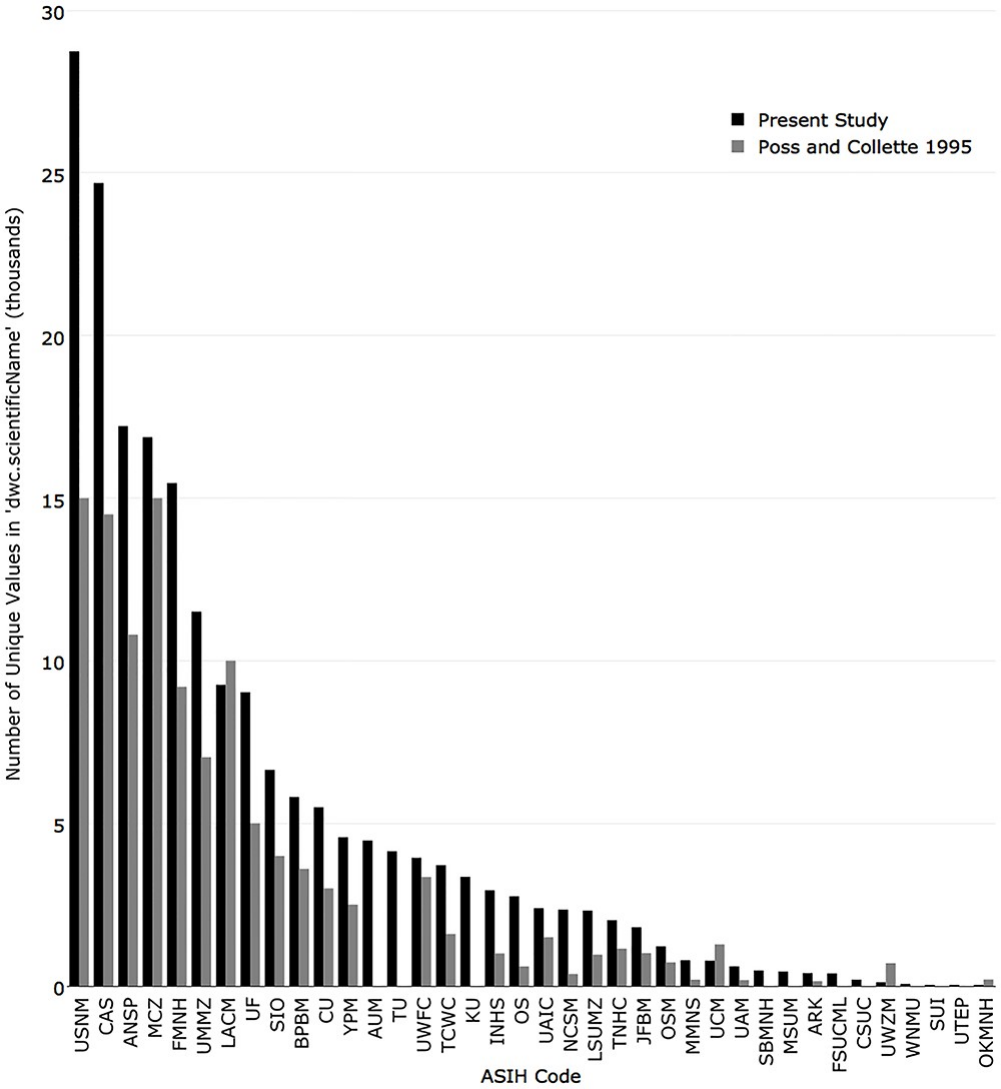
Comparison of the number of unique scientific name values currently published in iDigBio (black) with the number reported in Poss and Collette (1995) (grey).

In 1995, the five institutions with the most cataloged unique species name entries were National Museum of Natural History (15,000), Museum of Comparative Zoology, Harvard University (15,000), Academy of Natural Sciences of Drexel University (11,000), Natural History Museum of Los Angeles County (10,000), and Field Museum of Natural History (9,200). The institutions with the greatest change from 1995 were National Museum of Natural History (+13,742), California Academy of Sciences (+10,191), Academy of Natural Sciences of Drexel University (+6,415), Field Museum of Natural History (+6,266), and University of Michigan (+4,478) (Fig 10).

**Fig 11:**
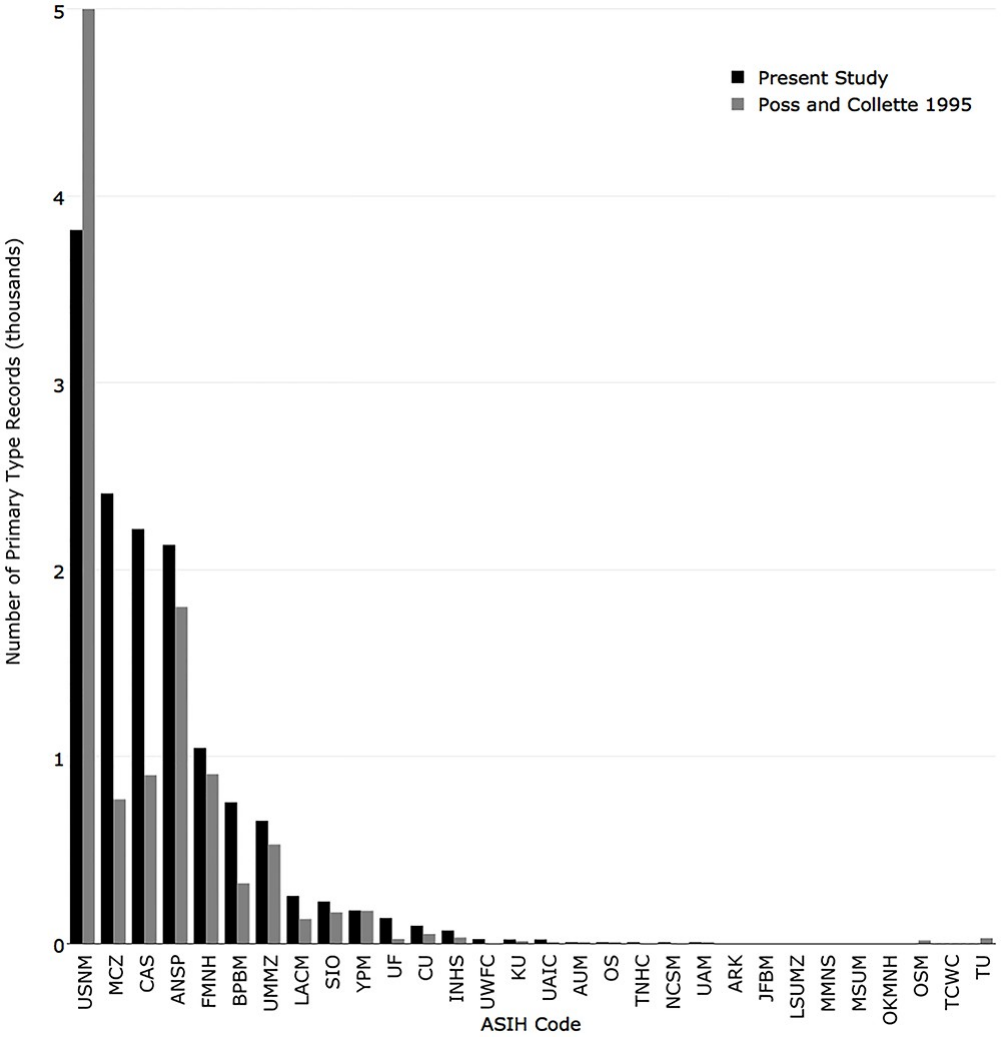
Comparison of the number of primary type records currently published in iDigBio (black) with the number reported in Poss and Collette (1995) (grey).

In 1995, the five institutions with the most primary types were National Museum of Natural History (5,000), Academy of Natural Sciences of Drexel University (1,800), Field Museum of Natural History (905), California Academy of Sciences (900), and Museum of Comparative Zoology, Harvard University (770). The institutions with the greatest change from 1995 were Museum of Comparative Zoology, Harvard University (+1,637), California Academy of Sciences (+1,317), National Museum of Natural History (-1,183), Bernice Pauahi Bishop Museum (+433), and Academy of Natural Sciences of Drexel University (+332). (Fig 11).

**Fig 12:**
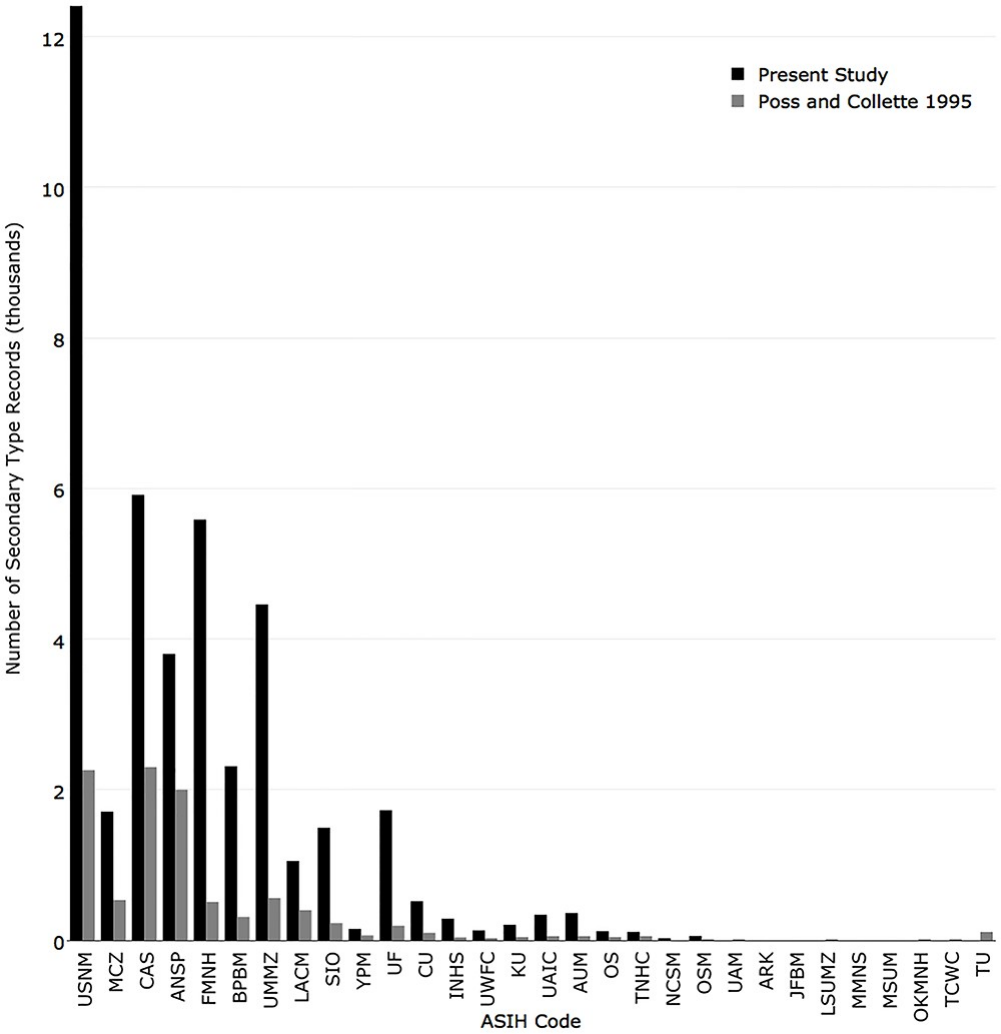
Comparison of the number of secondary type records currently published in iDigBio (black) with the number reported in Poss and Collette (1995) (grey).

In 1995, the five institutions with the most secondary type specimen records were California Academy of Sciences (2,300), National Museum of Natural History (2,260), Academy of Natural Sciences of Drexel University (2,000), University of Michigan (560), and Museum of Comparative Zoology, Harvard University (535). The institutions with the greatest change from 1995 were National Museum of Natural History (+10,148), Field Museum of Natural History (+5,076), University of Michigan (+3,899), California Academy of Sciences (+3,615), and Bernice Pauahi Bishop Museum (+2,000) (Fig 12).

**Fig 13:**
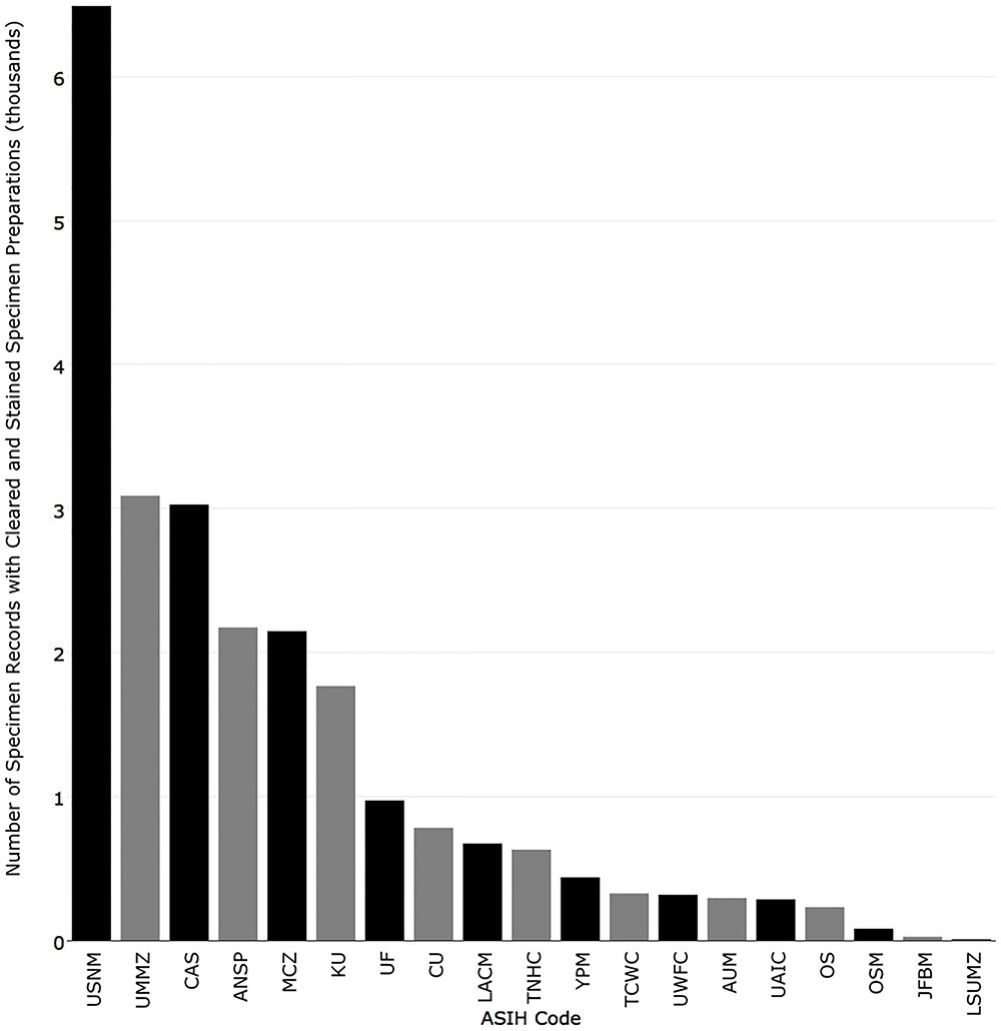
Number of records published in iDigBio with at least one cleared and stained specimen preparation.

**Fig 14:**
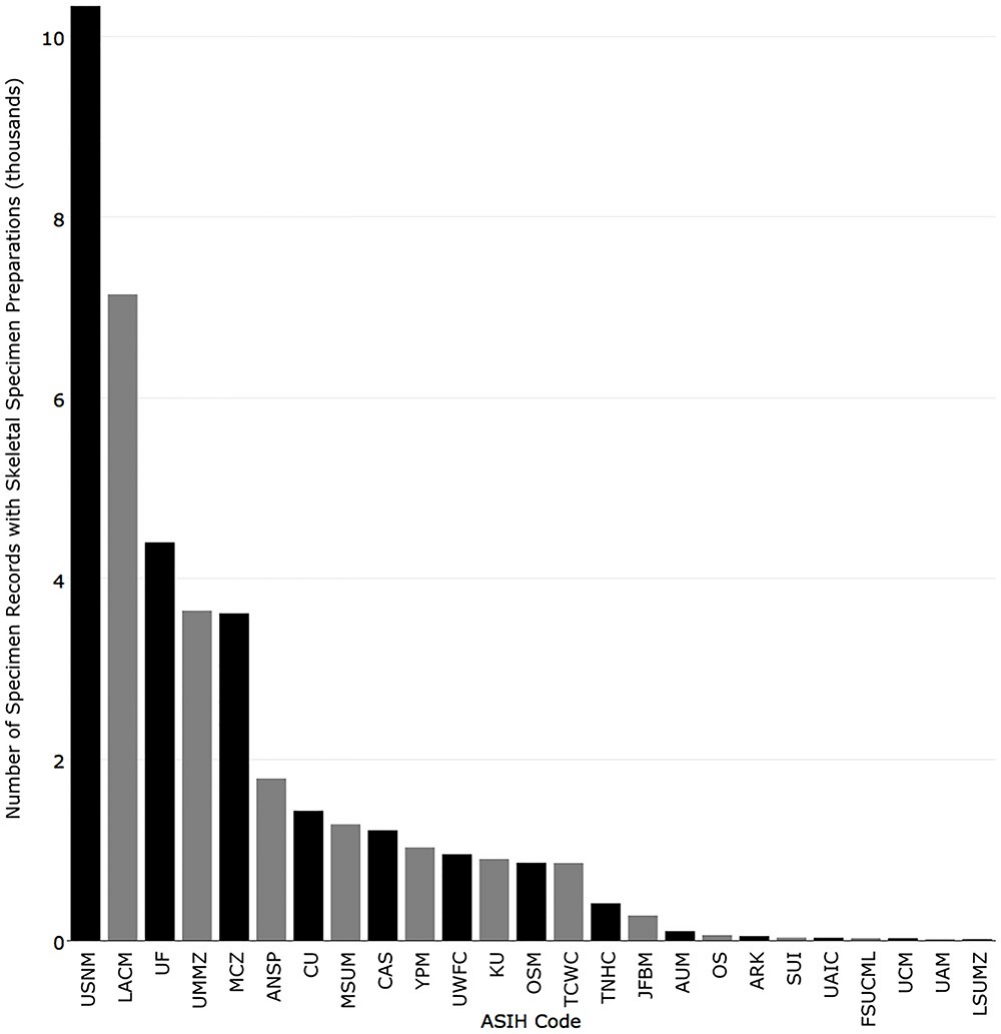
Number of records published in iDigBio with at least one skeletal specimen preparation.

**Fig 15:**
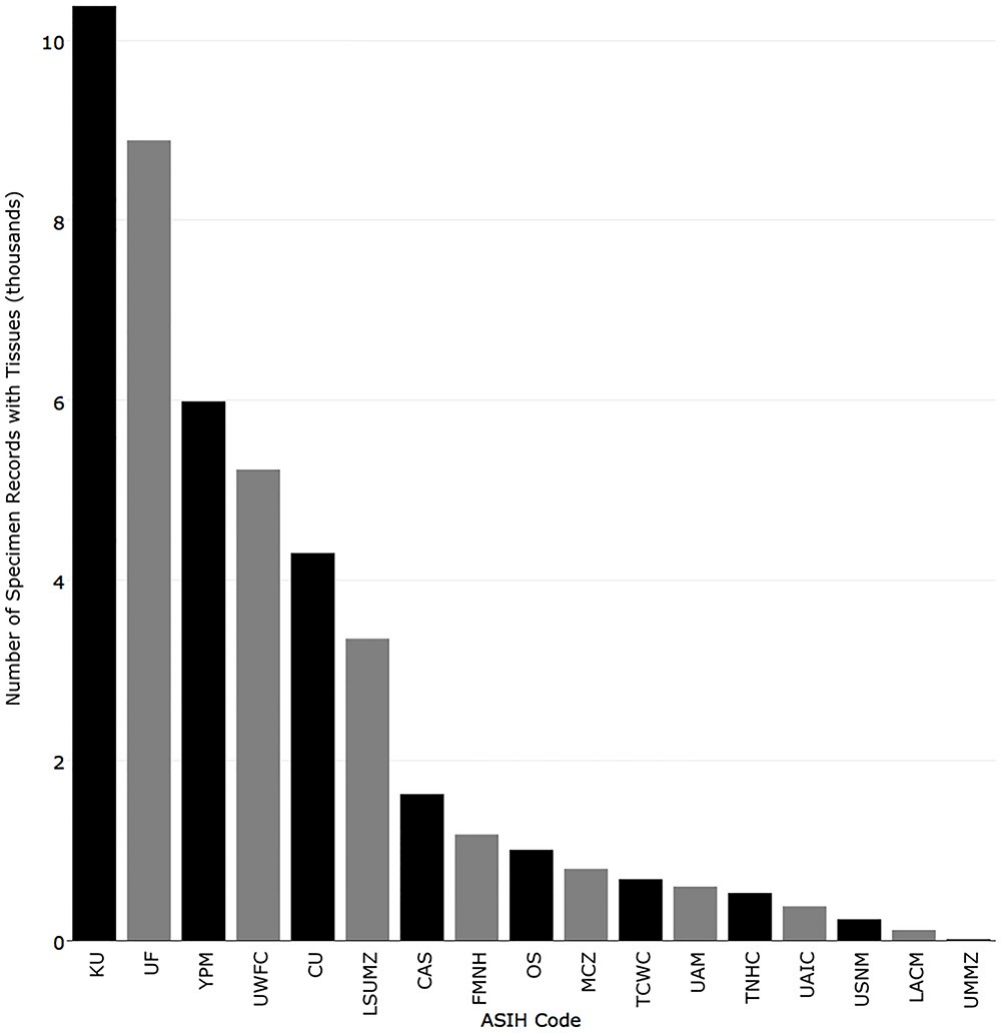
Number of records published in iDigBio with at least one tissue associated with a record.

Institutions with the most records that contain at least one cleared and stained specimen were National Museum of Natural History (6,490), University of Michigan (3,088), California Academy of Sciences (3,026), Academy of Natural Sciences of Drexel University (2,173), and Museum of Comparative Zoology, Harvard University (2,148) (Fig 13). Institutions with the most records that contain at least one skeletal specimen were National Museum of Natural History (10,337), Natural History Museum of Los Angeles County (7,147), Florida Museum of Natural History (4,402), University of Michigan (3,647), and Museum of Comparative Zoology, Harvard University (3,627) (Fig 14). Institutions with the most records that contain at least one tissue preserved for genetic analysis were Kansas University (10,381), Florida Museum of Natural History (8,887), Yale-Peabody Museum (5,988), University of Washington (5,229), and Louisiana Museum of Natural History (3,350) (Fig 15).

**Fig 16:**
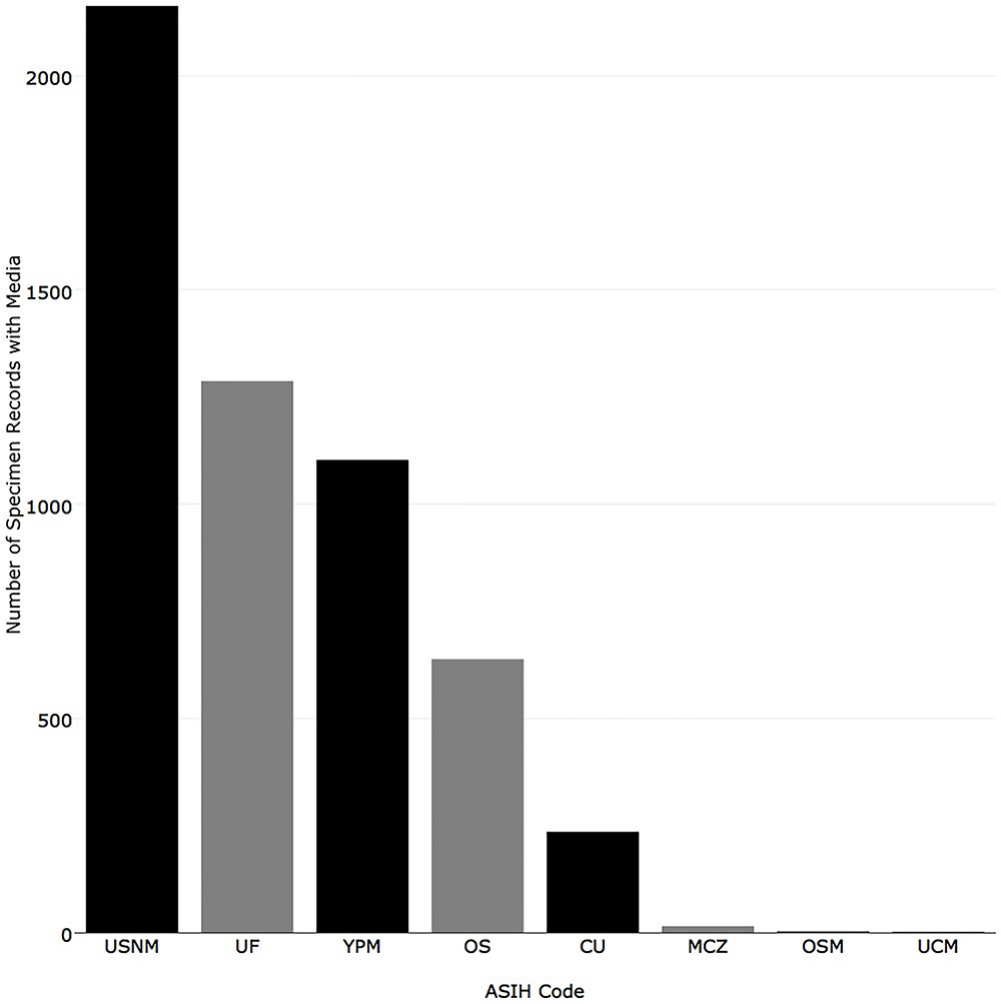
Number of records published in iDigBio with at least one type of media associated with a record.

**Fig 17:**
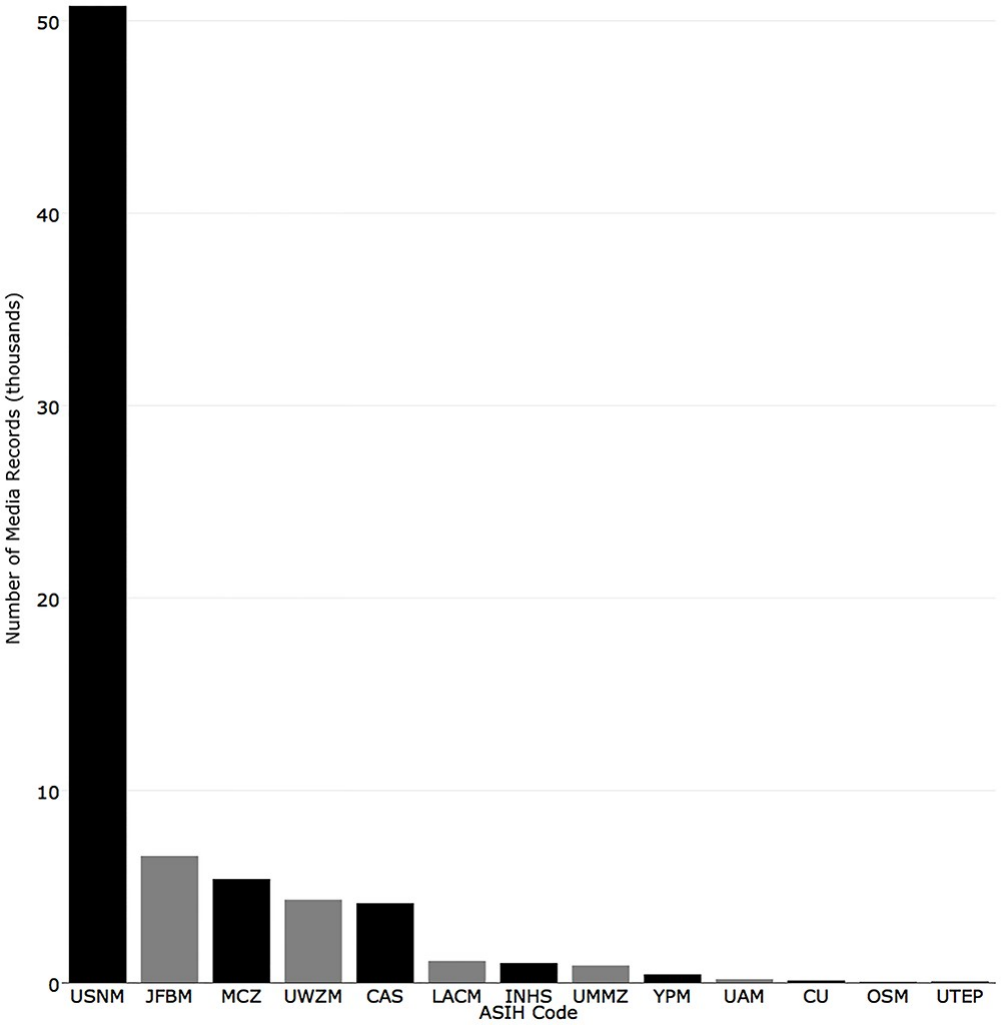
Number of media records associated with specimen records in iDigBio.

Two mechanisms for sharing information about media associated with specimen records (e.g., attached images or recordings) were examined. When collections recorded information about media in a record’s “preparation field,” these records were counted as having “media”. When a collection publishes media as a media record (e.g., an image), records were counted as “mediarecords”. Institutions with the most “media” were: National Museum of Natural History (2,163), Florida Museum of Natural History (1,287), Yale-Peabody Museum (1,103), Oregon State University (638), and Cornell University (235) (Fig 16). Institutions with the most mobilized “mediarecords” in iDigBio were National Museum of Natural History (50,769), Bell Museum of Natural History (6,575), Museum of Comparative Zoology, Harvard University (5380), University of Wisconsin Zoological Museum (4,301), and California Academy of Sciences (4,124) (Fig 17).

## Discussion

Poss and Collette [3] estimated institutional fish collection holdings based on information from collections’ staff. The present analysis explored data from institutional fish collections integrated in iDigBio, a data aggregator. In this strictly programmatic approach, data could be downloaded as desired without involving collections staff, and the data were easily updated from the aggregator as the analysis proceeded to give the most accurate snapshot of current collection holdings. This analysis provides a framework in which all digitized collections can be analyzed and compared using the ridigbio package and fishfindR web application.

Despite having data for only 38 fish collections, iDigBio contains information on the majority of fish specimens housed in U.S. collections. Eighteen of the 20 largest collections surveyed by Poss and Collette [3] were U.S. institutions, and 15 of those have mobilized data aggregated by iDigBio. Of the three largest U.S. collections included by Poss and Collette that have no data aggregated by iDigBio, The American Museum of Natural history (AMNH) had 200,000 lots; the University of Louisiana at Monroe (NLU) had 85,000 lots and Gulf Coast Research Laboratory (GCRL) had 36,000 lots. The NLU collection will eventually be incorporated into iDigBio when the specimens become integrated into the various collections to which they were rehoused following the loss of institutional support.

The programmatic nature of this analysis provided inherently more precise values for those collections that have mobilized their data and removed at least some of the inconsistencies associated with estimating collections holdings. For example, it is clear that several collections overestimated their holdings in 1995 (USNM, TCWC, OSM, MCZ, etc.) which puts them in an unfortunate position of not being able to demonstrate growth with some standard metrics (Figs 9 and 10)—even though it likely occurred. The overestimations were likely either due to several collections lacking a computerized database from which to generate reports or were based on estimates from collections staff that included large uncounted lots with overestimated or placeholder numbers (e.g., 9999) or the inclusion of uncataloged backlog material, which would not be found in iDigBio. Accurate data are important when reporting to administrators and funding agencies, and allow curators and collection managers to make informed decisions on how to optimize use and funding of their collections. Having specimens from poorly sampled taxa or geographic areas, or targeting such samples to add to a collection, can lead to increased use and heightened visibility of a collection. Collections that are not digitized are effectively invisible to much of the research community. In contrast, online access renders collections data accessible to all potential users, and visualization tools can be used to communicate valuable holdings to funding agencies, administrators, and the public.

Despite the approachability and ease of mobilizing data and the broadening community support for doing so, some institutional collections are not sharing their data for integration by a data aggregator. For the most part, collections that are not aggregated by iDigBio are small regional collections not associated with natural history museums, although some large institutions also are not mobilizing digitized data. Small institutions may be concerned that their data are less significant in the larger pool of collections data, but all collections have specimens of interest to researchers looking for comprehensive datasets. Collections staff also may be concerned that they need to mobilize data for multiple aggregators (e.g., GBIF and iDigBio); however, as a result of collaboration among aggregators, mobilizing data for one aggregator via shared tools such as IPT (Integrated Publishing Toolkit) results in data formatted to be mobilized to other aggregators. Also, mobilized data are now being shared among aggregators; e.g., data aggregated by VertNet go to iDigBio, and data aggregated by iDigBio go to GBIF.

Programmatic harvesting of collections data from iDigBio was not without complications. Collections data were not always structured uniformly when integrated by iDigBio, and some datasets had to be manually merged due to the nature of their data structure. Most institutions had a 1:1 ratio of recordset to collection; however, some institutions had many collections (e.g., fishes, amphibians, etc.) shared as one recordset, or several recordsets under one collection. An example of the latter was the University of Washington (UWFC). UWFC has 4 collections with unique names within the recordset: UWFC adult, UWFC juvenile, UWFC larval, UWFC egg. For this analysis, all of the collections were combined to give the entire holdings for the collection as was done for other institutions. Similarly, Kansas University (KU) maintains a tissue collection (KUIT) separate from its voucher specimens (KU). KUIT was excluded from the main analysis but was used to calculate the number of tissue preparations for that collection. In addition, some institutions have large collections (e.g., otoliths) that were not included in their digital database.

Another anomaly was found in the aggregated data from the Museum of Comparative Zoology, Harvard University (MCZ). At the time of data harvest, MCZ had a mobilization error in which the number of specimens was equal to the number of records. This error will be corrected in the web application when the data are corrected by the provider. Additionally some data of potential interest were not reported due to inconsistencies in how collections mobilize their data—in particular, not providing information in a particular field, such as number of specimens or taxonomic information. These gaps can be fixed by collections when they format their data according to the respective metadata standards for publication via a data aggregator.

Descriptions of the types of preparations (e.g., ethanol, tissue, and photograph) were not standardized across collections in the imported raw data, and some collections did not share preparation types in the correct field (dwc.preparations). Most of the null values can be assumed to be alcohol preservations, due to the default nature of fish specimens to be preserved in alcohol. While there were some collections data that used controlled vocabulary for preparations, such as “EtOH” and “skeleton”, many other terms, such as “churn” and “microdissection”, are open to interpretation.

Future work on collections might explore more in-depth specifics of collection infrastructure, particularly with regards to social aspects of collections similar to those found in Poss and Collette’s survey. Some examples might include institutional support, age of staff, graduate training and funding. These would need to be gathered via communication with collection staff, as they cannot be harvested from digitized collection records. In addition, some of the data from this survey might be further analyzed to compare and emphasize the importance of individual collections based on criteria such as taxonomic or preparation diversity. In addition, once collections begin using a unified data standard it might be easier to tease apart the details of preparations types in order to get information on large sub collections contained within some collections (e.g., large otolith collections disguised as “skeletal” or not discoverable due to the data being absent from the preparations field).

Using the tools provided via the ridigbio package and the framework provided herein, it is now possible to explore and to communicate about every collection that is digitized and aggregated within iDigBio. The data from these analyses can be used to answer many questions related to biodiversity, conservation, behavior, and life history, and this information on use can be given back to collections staff to aid in soliciting support. By exploring the data and increasing its transparency, aggregators and data providers can improve data quality. While sharing data via an aggregator is not the only means by which data can be shared, it is the most effective method of participating in a big data resource that is available to the largest assemblages of users [27]. The effectiveness of this type of resource incentivizes other collections to mobilize their data via data aggregators to increase data transparency, which in turn increases value and use as well as expands our information on biodiversity.

## Supporting information

S1 Appendix Summary data for fish collections arranged alphabetically by institutional code. (DOCX)
