# Supplementary material for: A survey of digitized data from U.S. fish collections in the iDigBio data aggregator
Source: PLoS One. 2018 Dec 19;13(12):e0207636. doi: 10.1371/journal.pone.0207636 (PMC6300206; doi:10.1371/journal.pone.0207636)
Supplement: S1 Appendix — (DOCX) [file pone.0207636.s001.docx]

**APPENDIX 1** - Summary data for fish collections arranged alphabetically by institutional code.

| **ASIH Code** | **Full Name** | **Management Staff** | **Number of records** | **Number of specimens** | **Number of primary types** | **Number of families:** |
| --- | --- | --- | --- | --- | --- | --- |
| ANSP | Academy of Natural Sciences of Drexel University – Ichthyology | *Curator:* Mark Sabaj  *Collection Manager:* Maria Arce Hernandez | 146,747 | 1,500,122 | 1,481 | 486 |
| UAFMC - ARK | University of Arkansas Collections Facility | *Curator:* Nancy McCartney | 5,850 | 157,858 | 0 | 90 |
| AUM | Auburn Museum of Natural History – Fishes | *Curator:* Jonathan Armbruster  *Collection Manager:* David Werneke | 66,982 | 728,806 | 5 | 296 |
| BPBM | Bernice Pauahi Bishop Museum – Ichthyology | *Curator:* Richard Pyle  *Collection Manager:* Arnold Suzumoto | 41,212 | 103,440 | 754 | 306 |
| CAS | California Academy of Natural Sciences – Ichthyology | *Curator:* Luis Rocha  *Collection Manager:* David Catania | 205,031 | 1,301,582 | 1,430 | 592 |
| CSUC | California State University, Chico Vertebrate Museum | *Curator:* Jay Bogiatto  *Collection Manager:* Emily Purvis | 408 | 2,212 | 0 | 59 |
| CUMV-CU | Cornell University Museum of Vertebrates – Fishes | *Curators:* Casey Dillman and William Bemis  *Collection Manager:* Charles M. Dardia | 90,894 | 1,253,589 | 94 | 385 |
| FMNH | The Field Museum – Fishes | *Collection Manager:* Caleb McMahan *Assistant Collection Managers:* Kevin Swagel and Susan Mochel | 106,196 | 851,742 | 1,045 | 539 |
| FSUCML | Florida State University Coastal and Marie Laboratory | *Curator:* Chip Cotton | 1,015 | 2,461 | 0 | 121 |
| INHS | Illinois Natural History Survey – Fishes | *Curator:* Christopher Taylor  *Collection Manager:* Dan Wylie | 109,283 | 881,318 | 68 | 237 |
| JFBM | Bell Museum of Natural History, University of Minnesota-Fishes | *Curator:* Andrew Simons | 38,769 | 335,200 | 0 | 234 |
| KU | Kansas University Biodiversity Institute and Natural History Museum – Ichthyology | *Curator:* Leo Smith  *Collection Manager:* Andrew Bentley | 41,314 | 526,619 | 20 | 406 |
| LACM | LA County Museum Fish Division | *Curator:* Christine Thacker  *Collection Manager:* Rick Feeney | 174,970 | 2,786,184 | 253 | 495 |
| LSUMZ | Louisiana Museum of Natural History – Fishes | *Curator:* Prosanta Chakrabarty | 17,672 | 329,902 | 0 | 289 |
| MCZ | Museum of Comparative Zoology, Harvard University Ichthyology | *Curator:* George Lauder  *Collection Manager:* Karsten Hartel | 172,899 | 172,899 | 2407 | 581 |
| MMNS | Mississippi Museum of Natural Science | *Curator:* Matt Wagner | 63,414 | 1,015,618 | 0 | 105 |
| MSUM | Michigan State University Museum | *Curator:* Michael Gottfried  *Collection Manager:* Laura Abraczinskas | 26,841 | NA | 0 | 178 |
| NCSM | North Carolina Museum of Natural Sciences - Ichthyology) | *Curator:* Alex Dornburg  *Collection Manager:* Gabriela Hogue | 80,128 | 1,976,129 | 2 | 278 |
| OKMNH | Sam Noble Museum | *Interim* *Curator:* Janet Braun  *Collection Manager:* Sara Cartwright | 56,250 | 1,978,306 | 0 | 45 |
| OS | Oregon State University, Department of Fisheries and Wildlife – Ichthyology Collection | *Curator:* Brian Sidlauskas  *Collection Manager:* Peter Konstantinidis | 19,219 | 130,775 | 4 | 362 |
| OSM | Ohio State University, Museum of Biological Diversity, Museum of Zoology | *Curator:* Marymegan Daly  *Associate Curator:* Marc Kibbey | 100,141 | 433,945 | 0 | 115 |
| SBMNH | Santa Barbara Museum of Natural History | *Curator:* Paul Collins  *Associate Curator:* Krista Fahy | 6,746 | 6,746 | 0 | 146 |
| SIO | Marine Vertebrate Collection of Scripps Institution of Oceanography | *Curator:* Philip Hastings  *Collection Manager:* Ben Frable | 115,846 | 1,382,653 | 223 | 497 |
| SUI | University of Iowa Museum of Natural History | *Collection Manager:* Cindy Opitz | 62 | 23 | 0 | 17 |
| TCWC | Biodiversity Research and Teaching Collections, Department of Wildlife and Fisheries Sciences, Texas A&M University | *Curators:* Kevin Conway and Heather Prestridge | 50,176 | 751,530 | 0 | 357 |
| TNHC | Texas Natural History Collections – Fishes | *Curator:* Dean Hendrickson  *Collection Manager:* Adam Cohen | 60109 | 1,350,695 | 3 | 260 |
| TU | Tulane University Museum of Natural History, Royal D. Suttkus Fish Collection | *Curator:* Henry Bart  *Collection Manager:* Justin Mann | 203,679 | 7,406,195 | 0 | 314 |
| UAIC | University of Alabama Ichthyology Collection | *Curator:* Phillip Harris  *Collection Manager:* Worth Pugh | 92,766 | 1,114,630 | 20 | 241 |
| UAM | University of Alaska Museum of the North | *Curator:* J. Andrés López | 9,968 | 47,468 | 0 | 96 |
| UCM | University of Colorado Museum of Natural History | *Curator:* Christy McCain  *Collection Manager:* Emily Braker | 2,271 | 25,040 | 0 | 163 |
| UF | Florida Museum of Natural History Fish Collection | *Curator:* Lawrence Page  *Collection Manager:* Robert Robins | 222,286 | 2,413,751 | 135 | 463 |
| UMMZ | University of Michigan Museum of Zoology | *Curator:* Hernán López-Fernandez  *Collection Manager:* Douglas Nelson | 197,376 | 3,461,252 | 655 | 461 |
| USNM | National Museum of Natural History Fish Collection | *Curator in Charge:* Dave Johnson  *Collection Manager:* Jeff Williams | 407,231 | 2,603,610 | 3,817 | 596 |
| UTEP | University of Texas at El Paso Ichthyology Collection | *Director:* Eli Greenbaum  *Collection Manager:* Teresa Mayfield | 98 | 1,322 | 0 | 11 |
| UWFC | University of Washington Fish Collection | *Curator:* Luke Tornabene  *Collection Manager:* Katherine Pearson-Maslenikov | 150,169 | 7,737,341 | 22 | 364 |
| UWZM | University of Wisconsin-Madison Zoological Museum | *Curator of Collections:* Laura Monahan  *Adjunct Curators*: Marlin Johnson and John Lyons | 4,601 | 57,170 | 0 | 15 |
| WNMU | Western New Mexico University | *Curator:* Randy Jennings | 379 | 1,690 | 0 | 10 |
| YPM | Yale Peabody Museum – Ichthyology | *Curator:* Thomas Near  *Collection Manager:* Gregory Watkins-Colwell | 28,339 | 263,385 | 175 | 411 |
